# Nuclear Envelope Regulation of Oncogenic Processes: Roles in Pancreatic Cancer

**DOI:** 10.3390/epigenomes2030015

**Published:** 2018-09-02

**Authors:** Claudia C. Preston, Randolph S. Faustino

**Affiliations:** 1 Genetics and Genomics, Sanford Research, Sioux Falls, SD 57104, USA;; 2 Department of Pediatrics, Sanford School of Medicine of the University of South Dakota, Sioux Falls, SD 57105, USA

**Keywords:** pancreatic cancer, nuclear envelope, nuclear lamina, LINC complex, nuclear pore complex, nucleoporins

## Abstract

Pancreatic cancer is an aggressive and intractable malignancy with high mortality. This is due in part to a high resistance to chemotherapeutics and radiation treatment conferred by diverse regulatory mechanisms. Among these, constituents of the nuclear envelope play a significant role in regulating oncogenesis and pancreatic tumor biology, and this review focuses on three specific components and their roles in cancer. The LINC complex is a nuclear envelope component formed by proteins with SUN and KASH domains that interact in the periplasmic space of the nuclear envelope. These interactions functionally and structurally couple the cytoskeleton to chromatin and facilitates gene regulation informed by cytoplasmic activity. Furthermore, cancer cell invasiveness is impacted by LINC complex biology. The nuclear lamina is adjacent to the inner nuclear membrane of the nuclear envelope and can actively regulate chromatin in addition to providing structural integrity to the nucleus. A disrupted lamina can impart biophysical compromise to nuclear structure and function, as well as form dysfunctional micronuclei that may lead to genomic instability and chromothripsis. In close relationship to the nuclear lamina is the nuclear pore complex, a large megadalton structure that spans both outer and inner membranes of the nuclear envelope. The nuclear pore complex mediates bidirectional nucleocytoplasmic transport and is comprised of specialized proteins called nucleoporins that are overexpressed in many cancers and are diagnostic markers for oncogenesis. Furthermore, recent demonstration of gene regulatory functions for discrete nucleoporins independent of their nuclear trafficking function suggests that these proteins may contribute more to malignant phenotypes beyond serving as biomarkers. The nuclear envelope is thus a complex, intricate regulator of cell signaling, with roles in pancreatic tumorigenesis and general oncogenic transformation.

## 1. Introduction

Pancreatic cancer is among the most aggressive forms of cancer in the world with a higher occurrence observed in developed countries [1,2]. In the United States, pancreatic cancer is the third most deadliest form of cancer, with a 5-year survival rate of only 8.5% and an incidence that has slowly but steadily risen in the past 15 years [3,4]. Exocrine pancreatic cancers, often located at the head of the pancreas, account for ≈95% of all diagnosed cases with the most common type being pancreatic ductal adenocarcinomas (PDAC). Other less common tumors, i.e., pancreatic neuroendocrine tumors, account for less than 5% of cases and have a more favorable 5-year survival rate at (50–65%) [2,5]. Still, PDAC is often synonymous for pancreatic cancer since it influences the vast majority of the epidemiological data of this disease.

PDAC is characterized by a large, firm mass with poorly defined margins and protrusions that extend inside the pancreas [6]. This aggressive tumor produces a strong desmoplastic reaction that contributes to its pervasive chemoresistance [7,8]. Gross pathology of PDAC biopsies typically present as whitish masses, that in conjunction with other distinctive histological features (i.e., presence of infiltrative but well-differentiated neoplastic glands, luminal necrosis, perineural, and vascular invasion), support the diagnosis of pancreatic adenocarcinoma [6]. PDAC elicits vague symptoms during early stages, with the most common being anorexia, fatigue, and weight loss; moreover, an increase in the interval from symptom onset to diagnosis has been observed in late stage exocrine pancreatic cancer [9–11].

There have been many established risk factors implicated in the risk of pancreatic cancer, including chronic diabetes mellitus, non-hereditary chronic pancreatitis, and some environmental risk factors, i.e., meat and fat high diets, obesity, alcohol, and tobacco use [12]. The genetic risk factors associated with pancreatic adenocarcinomas is a field that is in continuous growth and exploration [13–15]. Detailed in Table 1 are the most common somatic and germline mutations that have so far been detected in patients with PDAC. Dissecting the functional gene ontology of these genes implicates possible alternative pathways associated with pancreatic cancer that deserve exploration. The functional enrichment analysis of these common somatic and germline mutation genes contain functional terms common to both groups, being nucleoplasmic function and DNA regulation (Tables 2 and 3).

The development and progression of cancer has been attributed to: mitotic aberrations leading to aneuploidy; chromosomal abnormalities such as translocations, deletions, inversions, and duplications; and genome-wide chromothripsis [16]. Dysregulation of expression or regulation of tumor suppressors, oncogenes, and cell cycling controls can also initiate pathological signaling leading to oncogenesis [17–20]. Furthermore, epigenetic reader, writer, and eraser malfunction, with concomitant changes in chromatin structure, alters accessibility and disrupts proper programming [21]. Other nuclear regulatory modalities may play a role in cancer development given the emerging role of nuclear associated genes in pancreatic adenocarcinoma (Tables 2 and 3). Given the increased functional repertoire of the nuclear envelope in a variety of biological processes, nuclear envelope-related mechanisms of oncogenesis and their relevance to cancer biology are of critical importance and consideration.

## 2. Chromatin Organization and Dynamics

In general, chromatin access is highly regulated, underscored by dynamic rates of heterochromatin maintenance, and relaxation that control cellular processes in regular development and cancer [22]. Mechanisms of chromatin organization and accessibility include soluble nucleoplasmic machinery and nuclear envelope-bound components, with discrete temporospatial regulatory events that can occur within specific subnuclear domains [23–26]. For example, chromosome territories are the dynamic three-dimensional volumes occupied by each chromosome within the nucleus [27,28]. These non-static volumes are subject to spatial shifting in response to mitotic signaling [29,30], cellular aging cues [31], and DNA damage [32,33]. Chromosome territory dynamics facilitate gene positioning for transcriptional control [34], as well as gene triage for DNA repair [35]. Gene regulation also occurs within the spaces separating chromosome territories, known as interchromatin domains or compartments [36–38]. Within these spaces, post-transcriptional processing centers, such as nuclear speckles, can assemble to regulate transcriptional splicing [39,40]. This can occur throughout the nucleus, coordinating discrete chromosome territories to facilitate cis-and trans-regulation [40]. Heterochromatic compaction is subject to mechanisms that control chromosome dynamics. Cycling between heterochromatic relaxation and maintenance insures efficient ad hoc access to specific gene programs while simultaneously restricting access to inactive genes [41–43]. In concert with nucleosomal sliding and eviction, chromatin structural remodeling emerges as a highly dynamic system of global gene regulation and expression control. A key function of these chromatin dynamics is to bring super enhancers in contact with target genes, facilitating distal gene regulation [44–46]. This can be disrupted in cancer, where dysregulation of enhancer accessibility can promote malignancy [47,48].

Normal cellular function is dependent on proper maintenance of these various mechanisms of chromatin control, and their disruption has significant consequences for development and oncogenesis. The nuclear envelope interacts with this machinery and contributes to the regulation of chromatin structure and dynamics in addition to its function as the specialized intermediary between extranuclear and intranuclear compartments [49,50]. In this regard, the nuclear lamina, the LINC complex, and the nuclear pore complex emerge as key regulators of chromatin accessibility and cellular function, with relevant implications for cancer biology [51,52].

**Figure 1.**
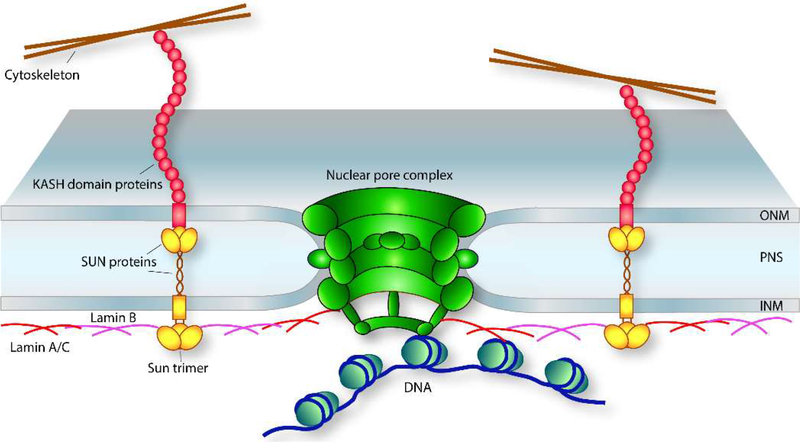
Landscape of the nuclear envelope. Schematic cross-section of a nuclear envelope illustrating the LINC complex, the nuclear lamina, and the nuclear pore complexes as they relate to one another. The LINC complex is connected to the cytoskeleton by the N-termini of the KASHdomain proteins with their C-termini embedded in the outer nuclear membrane (ONM) of the nuclear envelope. Within the perinuclear space (PNS), the KASH-domain C-term interacts with the C-term of the SUN protein within the inner nuclear membrane (INM). From there, the nucleoplasmic portion of the LINC complex connects to the nuclear lamina that interacts with chromatin as well as the nuclear pore complex. The nuclear lamina is located adjacent to the INM on the nucleoplasmic face where it forms a dynamic meshwork that provides several structural properties to the nucleus, i.e., nuclear stiffness regulated by composition of the nuclear lamina (see text); it serves to anchor nuclear pore complexes (NPCs) through interactions with distinct nucleoporins (nups) in the nuclear basket portion of the NPC; and it functions as a repressive subnuclear compartment. The NPC is a large multiproteinaceous complex that spans the nuclear envelope where the ONM and INM meet and is the main transporter that facilitates nucleocytoplasmic transport between nuclear and cytoplasmic compartments (see text).

As the barrier that physically and functionally sequesters chromatin from the cytoplasm, the nuclear envelope is the first regulatory entity that coordinates extranuclear stimulus with the nuclear response. It is a complex structure composed of two lipid bilayer systems, the inner nuclear membrane (INM) that immediately surrounds the chromatin, and the outer nuclear membrane (ONM) contiguous with the endoplasmic reticulum network (Figure 1). Interposed between the INM and ONM is the perinuclear space (PNS), which is functionally and topologically connected to the endoplasmic reticulum lumen [53].

## 3. The Nuclear Envelope

### 3.1. LINC Complexes

The linker of nucleoskeleton to cytoskeleton (LINC) complex mediates cytoskeletal communication with the nuclear interior [54]. “Tire! core of the LINC complex structure is the interaction between SUN-Sadh, UNC-84) and KASH (Klarsicht, ANC-1, Syne homology) domain proteins localized to the inner tINM) and outer (ONM) nuclear membranes of the nuclear envelope, respectively (Figure 1) [55]. The C-termini of both are located in the periplasmic space where they interact to transmit biomechanical stimuli from the cytoplasmic milieu into the nuclear interior to adjust biophysical properties of the nucleus. This interaction is critical to regulate nuclear positioning and deformability required for cellular migration [54]. In addition, the interactions between the C-termini of the SUN and KASH (domain proteins maintain uniformity of the periplasmic space [56]. As v gene expression regulator, SUN domain transmembrane proteins in the INM interact with the nuclear lamina via their N-terminuses [57], which in turn interact with nuclear .ore complexes and chromatin [58,59].

The KASH domain proteins consist primarily of nesprins that are found in the ONM, whereas SUN domain proteins are transported to the INM by diffusing from the ONM through the nuclear pore complex (NPC) to finally reside in the INM [60]. Upon proper localization, the interactions between the KASH and SUN domain proteins couple cytosolic microtubule, actin, and plectin networks to the nuclear interior [61–63], and are determinants of nuclear envelope thickness [64]. This distance ranges from 30–50 nm and is possible because of the varying C-terminal lengths of the different SUN protein isoforms [64]. Binding of SUN and KASH domain proteins can occur in varying ratios, with as many as three KASH proteins able to simultaneously bind one SUN trimer [65].

Recent work suggests the intriguing possibility that biomechanical forces transduced by the LINC complex are capable of directly regulating the nuclear transport function of the nuclear pore complex [66–68]. Interactions of SUN1 and SUN2 with nuclear export factor 1 (NXF1) as well as with components of the pre-export messenger ribonucleoprotein particle (mRNP) were disrupted following the SUN domain protein knockdown [68]. This caused mRNP accumulation within the nucleus, confirming the functional role of SUN domain proteins on nuclear export [68]. This wasfurther supported by evidence of SUN1 interactions with the nuclear basket nucleoporin NUP153, a critical component of the nuclear transport pathway [68].

Recent work suggests the intriguing possibility that biomechanical forces transduced by the LINC complex are capable of directly regulating the nuclear transport function of the nuclear pore complex [66–68]. Interactions of SUN1 and SUN2 with nuclear export factor 1 (NXF1) as well as with components of the pre-export messenger ribonucleoprotein particle (mRNP) were disrupted following the SUN domain protein knockdown [68]. This caused mRNP accumulation within the nucleus, confirming the functional role of SUN domain proteins on nuclear export [68]. This wasfurther supported by evidence of SUN1 interactions with the nuclear basket nucleoporin NUP153, a critical component of the nuclear transport pathway [68].

With respect to oncogenesis, it remains to be seen if the biophysical functions of the LINC complex contribute to oncogenic gene expression dysregulation per se, though reports suggest that cancer cell migration requires intact LINC complex structure and function [69–71]. Recent work by Infante et al. demonstrated that interactions between nesprin-2 and the dynein adaptor Lis1 coordinate with an extracellular matrix “digest-on-demand” mechanism to regulate cancer cell invasion. In this manner, while not a mechanism for initiating genomic catastrophe that precedes oncogenic transformation, LINC complex biology contributes to metastasis and persistence of malignancy.

### 3.2. Nuclear Lamina

The nuclear lamina is located adjacent to the inner nuclear membrane of the nuclear envelope (Figure 1) and regulates cell signaling by coordinating the assembly of multiple protein complexes [72–74]. It participates in chromatin organization, repressive transcriptional control, and DNA replication [75–77]. In addition, the nuclear lamina provides structural support to the nucleus [78]. The meshwork pattern observed for the lamina is composed of either A/C or B-type lamins that impart different physical properties to the lamin network [79]. Several integral INM proteins are associated with the nuclear lamina. In addition to SUN-domain proteins described above, LEM domain proteins (LAP2B, emerin, and MAN1), and lamin-b receptor (LBR) are associated with the nuclear lamina and contribute to gene expression regulation and nuclear form and function [80].

Nuclear deformability reflects lamin composition, with lamin A/C as the main lamin isoform that provides biomechanical stiffness to the nucleus [81,82]. In studies using cells lacking the A/C isoform, nuclei were fragile, less resistant to strain, and were frequently misshapen. In contrast, lamin B1 deficiency exhibited normal nuclear mechanics and biophysical properties despite increased nuclear blebbing. This led to the conclusion that lamin B1 was critical for nuclear integrity, but not stiffness. In support of this, micromanipulation of isolated nuclei to simulate physiological distension revealed that lamin A/C behaves as a polymeric shell with strain-stiffening properties that provide resistance to deformation [83]. A tunable auxetic property appears to be a dynamic function of the nuclear lamina [84,85]. At the extreme opposite end of this dynamism, cells can assemble a transient perinuclear actin network to mechanically rupture the lamina without damaging the nuclear envelope [86]. This facilitates cell deformability without cell death, making cells resilient as well as competent for migration.

In oncogenic models, a dysfunctional lamina is associated with pathological disruption of the nuclear envelope, which can lead to micronuclei formation that precedes genomic instability and aneuploidy [87–89]. Micronuclei possess transcriptional competency and may initiate chromothripsis, potentiating the development of cancer [90,91]. Indeed, morphological changes to the nuclear envelope, as well as micronuclei formation, have been used as criteria to diagnose or predict cancer [92]. For example, micronuclei detection in peripheral blood lymphocytes have been proposed to serves as predictive biomarkers for pancreatic cancer [93].

Nuclear scaling and size regulation is also impacted by lamins [94]. In general, an inverse relationship exists between lamin expression and nuclear size [95]. For example, depletion of lamin B1 increases the size of the lamina mesh and overall nuclear size in HeLa cells [96]. Abnormal lamin expression and/or localization that leads to enlarged nuclei is used as a cytopathological marker of cancer development and progression [52]. Thus, the nuclear lamina demonstrates roles in diagnosis and functional impacts on malignant growth, a characteristic exhibited by other NE components.

### 3.3. Nuclear Pore Complex

The NPC is a large multiproteinaceous structure that spans both membranes of the nuclear envelope (Figure 1) and regulates nucleocytoplasmic trafficking of molecules between nuclear and cytoplasmic compartments. The NPC is separated into three domains referred to as the cytoplasmic, central channel, and nucleoplasmic regions [97], and is composed of repetitive copies of ≈30 nuclear pore proteins, collectively termed nucleoporins. These are arranged in specific subcomplexes to form distinctive pore domains within each of the three regions [98,99]. These domains assemble to form a channel with a semipermeable hydrogel composed primarily of phenylalanine-glycine (FG) repeats [100,101]. A RanGTP/GDP cycle powers transport and acts in concert with soluble karyopherins to regulate molecular cargo transit [102].

NPC-mediated nuclear transport can drive cancer via mislocalization of cancer-related proteins [103] or through dysfunctional transport pathways. In general, a variety of import and export karyopherins, respectively termed importins and exportins, are overexpressed in a multitude of carcinomas [104]. This is advantageous as it offers candidates for therapeutic targeting [105–107], though rigorous characterization and testing are imperative to identify molecules with low to no adverse effects. Leptomycin B and its analog, selinexor, are inhibitors of exportin-1 (XPO1) that have been tested for their efficacy in treating pancreatic cancer [108,109]. Treatment of pancreatic cancer cell lines with these pharmacological inhibitors demonstrated effectiveness by deactivating NF-κB signaling and restoring tumor suppressive miR-145 [109,110]. In combination with gemcitabine, selinexor exhibited enhanced antitumor activity [111]. Recent identification of a novel karyopherin family member, KPNA7, is another potential target with pancreatic specificity. Initial reports identified overexpressed KPNA7 in multiple pancreatic cancer cell lines that implicated overactive transport of KPNA7 cargoes [112,113]. In an elegant series of experiments, Laurila et al. discovered that KPNA7 controlled pancreatic cancer cell proliferation by regulating p21 induction. This was supported by later work in which Vuorinen et al. demonstrated increased mitotic defects and nuclear deformation due to remodeling of the nuclear lamina and a shift in favor of lamin B1 [114].

Beyond anticipated roles in dysfunctional nuclear trafficking, a role for individual nucleoporins in cancer was discovered by the identification of nucleoporin fusion proteins in hematologic malignancies [115,116]. This early work identified fusions of NUP98 and NUP214 that led to a variety of de novo hematologic malignancies [117]. Further work identified other nups that promoted, as well as served as diagnostic markers, for a variety of other cancers [118–120]. To date, the individual nups associated with malignancy include NUP37, NUP88, NUP98, NUP160, NUP214, NUP358, and TPR [121–123].

NUP37 overexpression in a model of hepatocellular carcinoma was associated with enhanced metastasis and invasion that decreased upon NUP37 knockdown [123]. NUP88 was first identified as a marker for a variety of cancers, where its overexpression was diagnostic for tumorigenesis [119,120,124]. Recent identification of a phosphoregulatory function for NUP88 has been reported, where excess NUP88 inhibited dephosphorylation of vimentin in HeLa cells [118]. Dysregulation of the vimentin filament network can have consequences on pancreatic chemosensitivity, as vimentin is an active factor in the epithelial-to-mesenchymal transition that grants pancreatic tumors high chemoresistance [125,126]. NUP98, NUP160, and NUP214 have both been reported as fusion proteins with a variety of partners that lead to hematologic malignancies and angiosarcoma [117,122]. NUP358/RANBP2 also behaves as an oncogenic fusion protein [127], in addition to its transport associated mechanisms [128,129]. Novel functions for NUP358 were described in a recent report by Vecchione et al., where the authors discussed how NUP358 interacts with microtubules in a subset of colon cancer cells to promote their survival [130]. Similar to oncogenic mechanisms identified for the nups described above, TPR was identified in a variety of malignant fusion proteins that possessed constitutive tyrosine receptor kinase activity [131], whereas its overexpression promoted cancer cell survival through sustained mitosis. These examples demonstrate that a wide variety of mechanisms mediated by, or associated with nups can give rise to oncogenesis. Current work characterizing the expanding number and functional repertoire of nups that contribute to development may thus uncover novel mechanisms underlying general as well as tumor biology.

### 3.4. Potential Regulation of Epigenomic Dynamics by the Nuclear Envelope in the Setting of Pancreatic Cancer

An extant diversity of underlying epigenetic and epigenomic mechanisms drive pancreatic oncogenesis [132]. Among these, differential histone modifications can give rise to chromatin states that lead to the development of different subtypes of PDAC [21]. Methylation and acetylation states of various lysine residues on histone H3, i.e., H3K4, H3K9, and H3K27, as well as their localization within the nuclei, functionally and physically anchor discrete gene networks that establish pancreatic tumor aggression, metastasis, and resistance to therapy. The intimate relationship of histones, nuclear pore complexes, and nuclear lamina suggests a role for the nuclear envelope in regulating histone modification. This occurs in normal developmental processes mediated by lamina associated domains (LADs). Earlier work confirmed constitutive wild type LADs linked to transcriptional repression and histone H3 methylation [133,134]. This mechanism may have implications in chromatin remodeling that occurs in PDAC, as previous work identified lamin B1 overexpression in human pancreatic cancer associated with poor prognosis [135]. In pancreatic cancer cell models, lamin B1 knockdown significantly diminished proliferative aggressiveness and invasiveness [135]. Later independent work demonstrated that H3K27Me3 dispersed in a dramatic fashion from the nuclear periphery to the nuclear interior upon lamin B1 silencing [136], supporting the notion of the nuclear lamina playing a regulatory role in histone dynamics underlying oncogenesis.

Furthermore, the identification of enzymes such as histone deacetylases associated with individual nucleoporins supports the proposed regulatory roles of the nuclear envelope in histone modification and chromatin accessibility [137]. As an additional modality, NUP fusions to histone readers, as in the case of NUP98-PHD finger protein fusions, result in the “reading” of H3K4Me3 that locks associated genomic loci in an active state [138]. This type of mechanism is critical for hematologic transformation and establishes a rationale for its potential role in other cancer types. Indeed, the functional precedence for this system of H3K4Me3 detection and regulation exists for wild type NUP98 [139], and is significant when considering that H3K4Me3 is critical for anti-apoptotic gene activation in PDAC [140].

Epigenetic/epigenomic modifications and their association with the nuclear envelope in the setting of pancreatic oncogenesis is underscored in the “Triple Code Hypothesis,” where crosstalk among genetics, epigenetics, and nuclear structure plays an important role in the evolution of PDAC [141]. In support of this, the nuclear envelope’s regulatory contributions are mediated by the nuclear lamina, nucleoporins of the nuclear pore complex, and by the LINC complex. Evidence for the latter suggests that the LINC complexplays a larger role in metastatic dissemination and cancer metabolism over direct epigenomic regulation [70,142,143]. However, these biophysical and biochemical functions downstream of epigenomic remodeling furthermore do not preclude its potential primary contribution to oncogenic chromatin dynamics. This was recently demonstrated in work by Aymard et al., where the authors identify an active role for the LINC complex in clustering double-stranded DNA breaks induced in active genes [144]. This is particularly relevant as these gene lesions are critical for initiating and promoting tumorigenesis, and account for a major subtype of PDAC [145]. In addition, independent work showed that the expression of spectrin repeat containing nuclear envelope protein 2 (SYNE2/Nesprin-2), a component of the LINC complex, is dysregulated in PDAC by small nucleolar noncoding RNA SNORA23, and is associated with increased tumor invasion and metastasis in mice [146]. Given these initial findings, future studies are critical to define the role of the LINC complex in the context of tumorigenesis and PDAC.

## 4. Summary and Future Directions

While individual components of the nuclear envelope described above possess the capacity to regulate nuclear function in discrete ways, functional crosstalk among these components is critical to maintain a normal phenotype. The nuclear envelope thus emerges as an intricate regulatory body that integrates an array of biomechanical and biochemical signaling pathways to modulate nuclear structure and function. For example, developmental disruptions in any of the LINC complex, nuclear lamina, or NPC proteins are related to a class of diseases termed laminopathies that can affect multiple physiological systems [147]. This observation, in line with the enriched functional bias of known pancreatic cancer genes towards nucleoplasmic function and DNA regulation described earlier, supports the notion that the nucleus may have more roles in pancreatic cancer biology that are unexplored.

The high mortality rate associated with pancreatic cancer is a conflation of strong chemoresistance and high metastasis [148], and this underscores the priority to identify and develop specific and robust treatments with improved therapeutic potential. Exploiting the unique regulatory biology of the nuclear envelope may offer unique strategies for the design of novel therapeutic approaches to cancer. When considered in combination with extant treatment paradigms, such as cell cycle checkpoint inhibitors, oncolytic viruses, and pharmacological MEK antagonists [149], nuclear envelope interventions may contribute synergistic advantages to personalized cancer treatment regimens. Currently, immunotherapeutic approaches to pancreatic cancer are the most advanced paradigm being developed to address pancreatic malignancy [150] and involve the recruitment of activated T-cells. Adoptive T-cell therapy, or CAR-T cell therapy, has been promising in addressing hematologic malignancies [151,152] and has promise for treating pancreatic cancer. In this approach, chimeric antigen receptor (CAR) T-cells are autologously generated from extracted patient cells, then infused back into original donors. Despite the demonstrated success in a liquid tumor, challenges remain for its application to solid tumors. Progress in this field is ongoing, and it is anticipated CAR-T cell therapy has the potential to efficiently overcome the low immunogenicity of the pancreatic tumor microenvironment and facilitate its clearance and regression [149,153].

The functional priority of the nucleus associated with somatic mutations driving pancreatic malignancy provides the rationale to focus on nuclear elements that contribute to the pathology of pancreatic cancer. This is critical to developing novel strategies that synergize with current approaches to treat pancreatic malignancy. Thus, characterization of the nuclear envelope and its role with respect to oncogenesis will supplement the growing therapeutic armamentarium to address carcinomas and offers a body of knowledge that may be leveraged to overcome the innate resistance of pancreatic tumors.

## Figures and Tables

**Table 1.** Gene mutations associated with pancreatic adenocarcinoma [2].

Types of Mutation	Gene Symbol	Gene Description	Entrez Gene ID	Ensembl Gene ID
Somatic	*AKT2*	AKT serine/threonine kinase 2	208	ENSG00000105221
	*ARID1A*	AT-rich interaction domain 1A	8289	ENSG00000117713
	*BRAF*	B-Raf proto-oncogene, serine/threonine kinase	673	ENSG00000157764
	*CCSER1/FAM190A*	Coiled-coil serine rich protein 1	401,145	ENSG00000184305
	*CDKN2A*	Cyclin dependent kinase inhibitor 2A	1029	ENSG00000147889
	*EP300*	E1A binding protein p300	2033	ENSG00000100393
	*KMT2C/MLL3*	Lysine methyltransferase 2C	58,508	ENSG00000055609
	*KRAS*	KRAS proto-oncogene, GTPase	3845	ENSG00000133703
	*MYB*	MYB proto-oncogene, transcription factor	4602	ENSG00000118513
	*NCOA3/AIB1*	Nuclear receptor coactivator 3	8202	ENSG00000124151
	*SMAD4*	SMAD family member 4	4089	ENSG00000141646
	*TGFBR2*	Transforming growth factor beta receptor 2	7048	ENSG00000163513
	*TP53*	Tumor protein p53	7157	ENSG00000141510
	*USP9X*	Ubiquitin specific peptidase 9, X-linked	8239	ENSG00000124486
Germline	*ATM*	ATM serine/threonine kinase	472	ENSG00000149311
	*BRCA2*	BRCA2, DNA repair associated	675	ENSG00000139618
	*CDKN2A*	Cyclin dependent kinase inhibitor 2A	1029	ENSG00000147889
	*PALB2*	Partner and localizer of BRCA2	79,728	ENSG00000083093
	*PRSS1*	Protease, serine 1	5644	ENSG00000204983
	*STK11*	Serine/threonine kinase 11	6794	ENSG00000118046

**Table 2.** Functional enrichment analysis of common somatic genes mutated in pancreatic adenocarcinoma.

Cluster No. (E Score)	Database	Functional Term	Fold Enrichment	*p* Value	Benjamini
Cluster 1 (3.12)	UP_KEYWORDS	Acetylation	4.723	0.0000028	0.0002505
	KEGG_PATHWAY	Cell cycle	20.264	0.0006173	0.0033905
	UP_KEYWORDS	Transcription	4.904	0.0002617	0.0046472
	UP_KEYWORDS	Transcription regulation	5.043	0.0002192	0.0048656
	GOTERM_BP_DIRECT	Positive regulation of transcription, DNA-templated	15.049	0.0000176	0.0066071
	BIOCARTA	First Multivalent Nuclear Factor	28.889	0.0001714	0.0068348
	UP_KEYWORDS	Nucleus	2.803	0.0011438	0.0101343
	UP_KEYWORDS	Phosphoprotein	2.139	0.0013327	0.0107323
	GOTERM_CC_DIRECT	Nuclear chromatin	29.054	0.0002398	0.0112081
	GOTERM_CC_DIRECT	Nucleoplasm	4.028	0.0007497	0.0174706
	GOTERM_CC_DIRECT	Nucleus	2.589	0.0015626	0.0242026
	GOTERM_MF_DIRECT	DNA binding	5.430	0.0005138	0.0263695
Cluster 2 (2.95)	KEGG_PATHWAY	FoxO signaling pathway	28.128	0.0000006	0.0000098
	KEGG_PATHWAY	Adherens junction	26.543	0.0044414	0.0168064
	KEGG_PATHWAY	TGF-beta signaling pathway	22.435	0.0061682	0.0224314
Cluster 3 (2.86)	KEGG_PATHWAY	Chronic myeloid leukemia	61.073	0.0000000	0.0000000
	KEGG_PATHWAY	Pancreatic cancer	67.650	0.0000000	0.0000000
	KEGG_PATHWAY	Colorectal cancer	60.792	0.0000000	0.0000003
	KEGG_PATHWAY	HTLV-I infection	19.631	0.0000000	0.0000003
	KEGG_PATHWAY	Pathways in cancer	12.787	0.0000002	0.0000037
	KEGG_PATHWAY	FoxO signaling pathway	28.128	0.0000006	0.0000098
	KEGG_PATHWAY	Non-small cell lung cancer	56.088	0.0000008	0.0000111
	KEGG_PATHWAY	Glioma	48.322	0.0000014	0.0000178
	KEGG_PATHWAY	Melanoma	44.238	0.0000021	0.0000226
	KEGG_PATHWAY	Prostate cancer	35.692	0.0000049	0.0000482
	KEGG_PATHWAY	Thyroid hormone signaling pathway	27.552	0.0000137	0.0001230
	KEGG_PATHWAY	Bladder cancer	61.286	0.0000226	0.0001865
	KEGG_PATHWAY	Hepatitis B	21.661	0.0000354	0.0002697
	KEGG_PATHWAY	Endometrial cancer	48.322	0.0000465	0.0003286
	KEGG_PATHWAY	Renal cell carcinoma	38.657	0.0000910	0.0006002
	UP_KEYWORDS	Disease mutation	5.188	0.0000396	0.0011754
	KEGG_PATHWAY	MAPK signaling pathway	12.317	0.0003191	0.0019727
	KEGG_PATHWAY	Neurotrophin signaling pathway	20.939	0.0005607	0.0032611
	KEGG_PATHWAY	Hepatitis C	18.893	0.0007577	0.0037452
	KEGG_PATHWAY	Thyroid cancer	64.984	0.0007496	0.0038995
	UP_KEYWORDS	Proto-oncogene	24.916	0.0003911	0.0043423
	KEGG_PATHWAY	Proteoglycans in cancer	12.564	0.0024661	0.0115730
	KEGG_PATHWAY	Viral carcinogenesis	12.257	0.0026465	0.0118544
	KEGG_PATHWAY	Acute myeloid leukemia	33.653	0.0027845	0.0119306
	KEGG_PATHWAY	Central carbon metabolism in cancer	29.446	0.0036227	0.0148594
	KEGG_PATHWAY	Long-term potentiation	28.554	0.0038486	0.0151538
	UP_SEQ_FEATURE	Mutagenesis site	5.233	0.0001733	0.0202413
	KEGG_PATHWAY	MicroRNAs in cancer	8.817	0.0067131	0.0227320
	KEGG_PATHWAY	ErbB signaling pathway	21.661	0.0066041	0.0231553
	KEGG_PATHWAY	Progesterone-mediated oocyte maturation	21.661	0.0066041	0.0231553
	GOTERM_CC_DIRECT	Cytosol	3.383	0.0021984	0.0255285
	UP_KEYWORDS	Isopeptide bond	6.493	0.0043625	0.0274111
	UP_KEYWORDS	Apoptosis	10.971	0.0041342	0.0279639
	KEGG_PATHWAY	PI3K-Akt signaling pathway	7.283	0.0113878	0.0370899
	KEGG_PATHWAY	Sphingolipid signaling pathway	15.705	0.0122869	0.0387127
	UP_KEYWORDS	Kinase	8.000	0.0099232	0.0434085
	KEGG_PATHWAY	Signaling pathways regulating pluripotency of stem cells	13.461	0.0164870	0.0486500
	KEGG_PATHWAY	Insulin signaling pathway	13.656	0.0160424	0.0488026
Cluster 4 (2.60)	KEGG_PATHWAY	Cell cycle	20.264	0.0006173	0.0033905
	KEGG_PATHWAY	Viral carcinogenesis	12.257	0.0026465	0.0118544
	KEGG_PATHWAY	MicroRNAs in cancer	8.817	0.0067131	0.0227320
	GOTERM_MF_DIRECT	p53 binding	58.144	0.0009983	0.0340310
	GOTERM_BP_DIRECT	Apoptotic process	11.391	0.0005128	0.0470735
Cluster 5 (2.58)	KEGG_PATHWAY	Hepatitis B	21.661	0.0000354	0.0002697
	KEGG_PATHWAY	Cell cycle	20.264	0.0006173	0.0033905
	UP_KEYWORDS	Ubl conjugation	6.035	0.0003300	0.0048835
	GOTERM_BP_DIRECT	Negative regulation of transcription from RNA polymerase II promoter	10.764	0.0000880	0.0164117
	UP_KEYWORDS	Isopeptide bond	6.493	0.0043625	0.0274111
	GOTERM_BP_DIRECT	Positive regulation of transcription from RNA polymerase II promoter	7.900	0.0003776	0.0462357
	KEGG_PATHWAY	Wnt signaling pathway	13.656	0.0160424	0.0488026
Cluster 6 (1.94)	UP_KEYWORDS	Acyltransferase	25.791	0.0050407	0.0295385

Functional enrichment analysis was performed with DAVID Bioinformatics Resources 6.8 using high stringency. Depicted are the functional clusters, with their enrichment scores (E scores), comprised of statistically significant terms (*p* < 0.05).

**Table 3.** Functional enrichment analysis of germline genes mutated in pancreatic adenocarcinoma.

Cluster No. (E Score)	Database	Functional Term	Fold Enrichment	*p* Value	Benjamini
Cluster 1 (4.25)	GOTERM_BP_DIRECT	Strand displacement	322.923	0.0000230	0.0038316
	GOTERM_BP_DIRECT	DNA synthesis involved in DNA repair	239.886	0.0000420	0.0035042
	GOTERM_BP_DIRECT	Double-strand break repair via homologous recombination	113.459	0.0001900	0.0105193
Cluster 2 (2.74)	UP_KEYWORDS	Tumor suppressor	96.897	0.0000000	0.0000015
	GOTERM_CC_DIRECT	Nucleoplasm	5.455	0.0023864	0.0557288
Cluster 3 (1.76)	GOTERM_BP_DIRECT	Cell cycle arrest	59.546	0.0006886	0.0283490
Cluster 4 (1.72)	UP_KEYWORDS	Cell cycle	21.109	0.0002990	0.0056659

Functional enrichment analysis was performed with DAVID Bioinformatics Resources 6.8 using high stringency. Depicted are the functional clusters, with their enrichment scores (E scores), comprised of statistically significant terms (*p* < 0.05).
